# The Flavonol Quercitrin Hinders GSK3 Activity and Potentiates the Wnt/β-Catenin Signaling Pathway

**DOI:** 10.3390/ijms232012078

**Published:** 2022-10-11

**Authors:** Danilo Predes, Lorena A. Maia, Isadora Matias, Hannah Paola Mota Araujo, Carolina Soares, Fernanda G. Q. Barros-Aragão, Luiz F. S. Oliveira, Renata R. Reis, Nathalia G. Amado, Alessandro B. C. Simas, Fabio A. Mendes, Flávia C. A. Gomes, Claudia P. Figueiredo, Jose G. Abreu

**Affiliations:** 1 Instituto de Ciências Biomédicas, Universidade Federal do Rio de Janeiro, Rio de Janeiro 21941-902, Brazil; 2 Faculdade de Farmácia, Universidade Federal do Rio de Janeiro, Rio de Janeiro 21941-901, Brazil; 3 Instituto de Pesquisas de Produtos Naturais Walter Mors, Universidade Federal do Rio de Janeiro, Rio de Janeiro 21941-901, Brazil

**Keywords:** flavonoid, quercetin glycoside, Wnt signaling, GSK3 phosphorylation, Alzheimer disease

## Abstract

The Wnt/β-catenin signaling pathway dictates cell proliferation and differentiation during embryonic development and tissue homeostasis. Its deregulation is associated with many pathological conditions, including neurodegenerative disease, frequently downregulated. The lack of efficient treatment for these diseases, including Alzheimer’s disease (AD), makes Wnt signaling an attractive target for therapies. Interestingly, novel Wnt signaling activating compounds are less frequently described than inhibitors, turning the quest for novel positive modulators even more appealing. In that sense, natural compounds are an outstanding source of potential drug leads. Here, we combine different experimental models, cell-based approaches, neuronal culture assays, and rodent behavior tests with *Xenopus laevis* phenotypic analysis to characterize quercitrin, a natural compound, as a novel Wnt signaling potentiator. We find that quercitrin potentiates the signaling in a concentration-dependent manner and increases the occurrence of the *Xenopus* secondary axis phenotype mediated by *Xwnt8* injection. Using a GSK3 biosensor, we describe that quercitrin impairs GSK3 activity and increases phosphorylated GSK3β S9 levels. Treatment with XAV939, an inhibitor downstream of GSK3, impairs the quercitrin-mediated effect. Next, we show that quercitrin potentiates the Wnt3a-synaptogenic effect in hippocampal neurons in culture, which is blocked by XAV939. Quercitrin treatment also rescues the hippocampal synapse loss induced by intracerebroventricular injection of amyloid-β oligomers (AβO) in mice. Finally, quercitrin rescues AβO-mediated memory impairment, which is prevented by XAV939. Thus, our study uncovers a novel function for quercitrin as a Wnt/β-catenin signaling potentiator, describes its mechanism of action, and opens new avenues for AD treatments.

## 1. Introduction

The Wnt/β-catenin signaling pathway dictates cell proliferation and differentiation during embryonic development and tissue homeostasis [1]. Deregulation of Wnt/β-catenin signaling is associated with birth defects, cancer, and degenerative diseases [2]. In the central nervous system (CNS), the Wnt pathway plays a role in synaptic transmission [3] and plasticity [4], and its activation enhances long-term potentiation (LTP), while its inhibition impairs LTP [5]. In addition, suppressed canonical Wnt signaling plays a role in Alzheimer’s disease (AD) [6,7,8], an age-related neurodegenerative disease. Brain deposition of neuritic plaques and neurofibrillary tangles containing β-amyloid and phospho-tau (p-tau) proteins are hallmarks of AD [9]. Amyloid-β oligomers (AβO) are the main neurotoxins found in AD brains [10], and their accumulation correlates with cognitive decline [11] and LTP impairment [12]. Wnt/β-catenin signaling suppression leads to increased Aβ-induced neuronal apoptosis [13]. In contrast, canonical Wnt signaling activation with lithium [14], andrographolide [15], or potentiation with WASP-1 (Wnt-activating small molecule potentiator-1) [16] enhances cognitive function and reverses AD cognitive deficits. Mechanistically, Wnt signaling improves AD pathogenesis by suppressing tau phosphorylation via GSK3β [17], impairing Aβ aggregation via downregulation of β-site APP cleaving enzyme (BACE1) [18], enhancing synaptic plasticity [4], and promoting neurogenesis [19]. Therefore, Wnt signaling activation is a suitable strategy for AD treatment.

The absence of efficient treatment for AD inspires the quest for novel therapeutic candidates [20]. In this context, plant-derived compounds have been used for medicinal purposes since 2900 BC [21] and remain the most reliable source of potential drug leads [22]. Among the various plant-derived secondary metabolites, epidemiological studies have noticed the beneficial properties of flavonoids in preventing neuronal death, synaptic failure, and neurodegenerative diseases [23]. Furthermore, some flavonoids, such as fisetin [24], 7,8-Dihydroxyflavone [25], green tea catechins [26], and hesperidin [27], improve synapse formation. Additionally, flavonoids display many biological effects such as anti-oxidative, anti-inflammatory, anti-viral, and antitumoral effects while modulating different signaling pathways [28,29,30].

We have encountered quercitrin (quercetin-3-*O*-α-l-rhamnoside), a quercetin derivative, through a natural compounds cell-based screening as a Wnt/β-catenin signaling positive modulator. Quercitrin can be commonly found in food, beverages, and in traditional Chinese medicine [31]. Traditionally, quercitrin has been isolated from the bark of *Quercus tinctoria*, from lemon flavine, from the flowers of *Albizia julibrissin* Durazz, and from the tartary buckwheat (*Fagopyrum tataricum* [L.] Gaertn.) [31]. Quercitrin has anti-inflammatory [32], antileishmanial [33], osteogenic [34] and antinociceptive properties [35]. Our group and others have shown quercetin [36,37] and isoquercitrin [38,39] as Wnt/β-catenin signaling inhibitors. Quercetin, isoquercitrin, and quercitrin chemical structure similarity and their differential effects on Wnt signaling modulation, combined with the need to characterize novel Wnt signaling activators, motivated the elucidation of the mechanism underlying the action and functional effects of quercitrin.

In the present work, we combine cell-based assays with in vivo approaches using different vertebrate animal models, including mice and *Xenopus laevis*. We used *Xenopus* early development, a gold standard canonical Wnt signaling model, to describe quercitrin as a Wnt/β-catenin signaling potentiator that hinders Glycogen Synthase Kinase-3 (GSK3) kinase activity. Moreover, quercitrin potentiates the canonical Wnt synaptogenic effect in hippocampal neuronal cultures and rescues AβO-induced memory impairment in mice. Overall, we characterize quercitrin as a Wnt/β-catenin potentiator and a promising therapeutic candidate for treating AD.

## 2. Results

### 2.1. Quercitrin Potentiates the Wnt/β-Catenin Signaling Pathway

**Figure 1:**
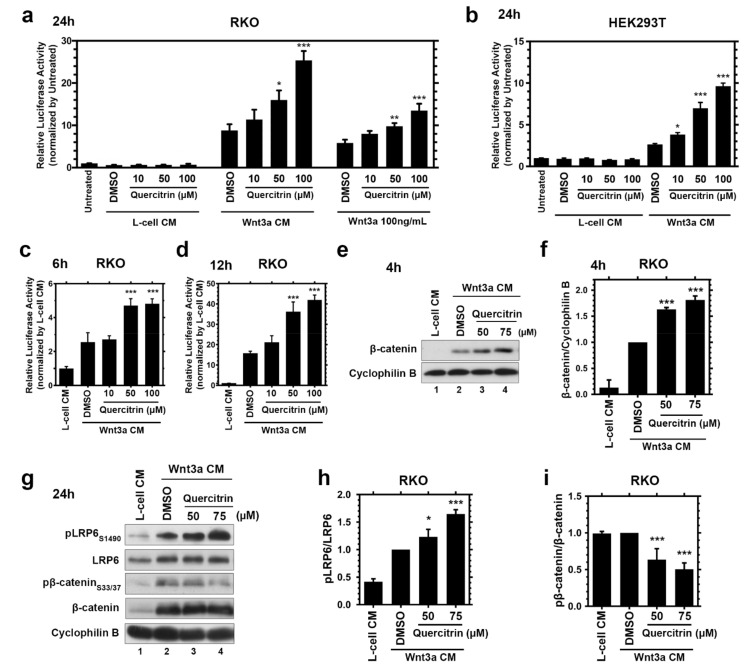
Quercitrin potentiates the Wnt/β-catenin signaling pathway. (**a**) Reporter gene assay in RKO B/R cells treated with L-cell CM, Wnt3a CM or recombinant human Wnt3a. The cells were treated with the vehicle dimethyl sulfoxide (DMSO) or quercitrin for 24 h *(n = 3*, performed in triplicate, one-way ANOVA followed by Dunnett’s multiple comparisons test, * p < 0.05, ** p < 0.01, *** p < 0.001). Error bars denote mean ± SD. (**b**) TOPFLASH reporter gene assay in transfected HEK293T cells treated with L-cell CM or Wnt3a CM. The cells were treated with the vehicle DMSO or quercitrin for 24 h *(n = 3*, performed in triplicate, one-way ANOVA followed by Dunnett’s multiple comparisons test, * p < 0.05, *** p < 0.001). Error bars denote mean ± SD. (**c**) Reporter gene assay of RKO B/R cells treated for 6 h *(n = 3*, performed in triplicate, one-way ANOVA followed by Dunnett’s multiple comparisons test, *** p < 0.001). Error bars denote mean ± SD. (**d**) Reporter gene assay of RKO B/R cells treated for 12 h *(n =* 3, performed in triplicate, one-way ANOVA followed by Dunnett’s multiple comparisons test, *** p < 0.001). Error bars denote mean ± SD. (**e**) Immunoblot assay of RKO cells lysate after 4 h treatment. RKO cells were treated with L-cell CM (lane 1) or Wnt3a CM (lanes 2–4). (**f**) Densitometric analysis of (**e**) showing that quercitrin increases β-catenin protein levels *(n =* 4, one-way ANOVA followed by Dunnett’s multiple comparisons test, *** p < 0.001). Error bars denote mean ± SD. (**g**) Immunoblotting of RKO cells treated for 24 h. RKO cells were treated with L-cell CM (lane 1) or Wnt3a CM (lanes 2–4). (**h**) Densitometric analysis of (**g**) pLRP6/LRP6 protein levels revealing that quercitrin increases pLRP6 S1490 and decreases pβ-catenin S33/S37 protein levels *(n =* 4, performed in triplicate, one-way ANOVA followed by Dunnett’s multiple comparisons test, * p < 0.05, *** p < 0.001). Error bars denote mean ± SD. (**i**) Densitometric analysis of (**g**) pβ-catenin/β-catenin protein levels revealing that quercitrin decreases pβ-catenin S33/S37 protein levels *(n =* 4, one-way ANOVA followed by Dunnett’s multiple comparisons test, *** p < 0.001). Error bars denote mean ± SD.

Through a cell-based screening of natural compounds, we noticed that quercitrin increases the Wnt/β-catenin signaling activity. Considering that Wnt signaling is a well-conserved signaling pathway across the various eukhariotic cells [1], we studied the modulation of its activity employing the colorectal cell line RKO, which displays a robust Wnt signaling activation due to its low basal Wnt activity [40]. To elucidate this modulation, we treated RKO B/R cells, a Wnt/β-catenin reporter gene containing cell line [41], with L-cell conditioned medium (CM), Wnt3a CM, or recombinant 100 ng/mL recombinant hWnt3a with 10, 50, or 100 μM quercitrin for 24 h. Reporter gene assays showed that quercitrin treatment, without Wnt3a CM or recombinant human Wnt3a (hWnt3a), does not activate the canonical Wnt signaling (Figure 1a). Different concentrations of quercitrin enhanced the Wnt signaling activation induced by either Wnt3a CM or recombinant hWnt3a. Quercitrin potentiated the effect of Wnt3a CM close to 1.8-fold at 50 μM and 2.9-fold at 100 μM. While RKO cells treated with hWnt3a and quercitrin displayed an increase of 1.7-fold at 50 μM and 2.3-fold at 100 μM compared to vehicle conditions. HEK293T transfected with TOPFLASH reporter assayed with the same concentrations of quercitrin and treated with L-cell CM or Wnt3a CM displayed similar results (Figure 1b). Quercitrin at 50 μM and 100 μM potentiated the Wnt signaling activation by 2.6-fold and 3.6-fold, respectively. In addition, quercitrin potentiated the Wnt signaling in a concentration dependent-manner in HEK293T cells transfected with increasing concentrations of hWnt3a plasmid (Figure S1). Quercitrin also potentiated canonical Wnt reporter gene activation when cells were treated for 6 h, leading to a 1.9-fold increase (Figure 1c), or for 12 h, leading to a 2.7-fold increase (Figure 1d). These results suggest that quercitrin does not activate the Wnt signaling pathway but potentiates it.

Canonical Wnt signaling functions through β-catenin degradation regulation. GSK3 and CK1, two kinases present in the destruction complex, also composed of Axin and APC, phosphorylate β-catenin, marking it for proteasomal degradation. The presence of Wnt ligands triggers the destruction complex recruitment to the membrane in a dynamic manner leading to β-catenin stabilization and target genes transcription [1,42,43]. RKO cells are a convenient platform to access beta-catenin stabilization since these cells do not have membrane-bound β-catenin [44]. Thus, the analysis of the β-catenin protein levels is a valuable readout of Wnt signaling activity. The immunoblot assay showed that 4 h quercitrin treatment of RKO cells together with Wnt3a CM increased β-catenin stabilization (Figure 1e,f). To further verify the quercitrin potentiation effect, the phosphorylation levels of key components of the Wnt/β-catenin pathway were measured through immunoblotting. Quercitrin and Wnt3a CM co-treatment increased the phosphorylation levels of LRP6 S1490 and reduced the phosphorylation of β-catenin S33/S37, hallmarks of Wnt pathway activation, thus corroborating the signaling pathway potentiation mechanism (Figure 1g–i).

**Figure 2:**
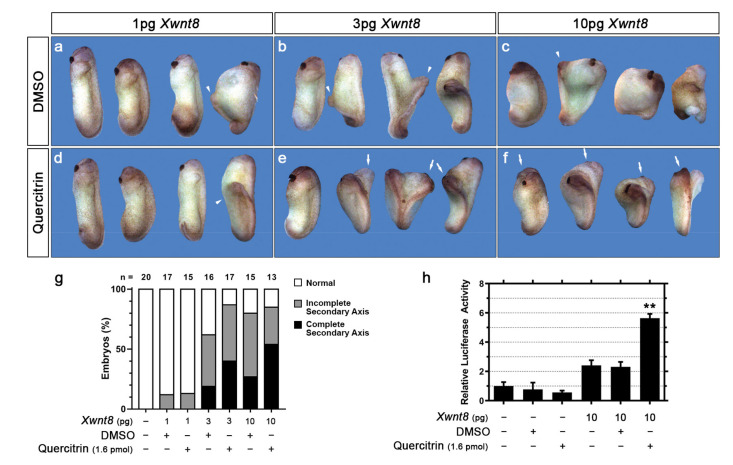
Quercitrin potentiates the Wnt/β-catenin signaling pathway in *Xenopus laevis*. (**a**–**f**) Representative embryos injected with increasing concentrations of *xWnt8* mRNA into the ventral blastomere, co-injected with DMSO or 1.6 pmol quercitrin. The injection induces the formation of an incomplete (arrow head) or a complete secondary axis (arrow). (**g**) Phenotype quantification of (**a**–**f**). The coinjection with quercitrin increases the complete secondary axis phenotype occurrence. The number of embryos in each condition is shown above the graph bars. (**h**) S01234 reporter gene assay. Quercitrin coinjection potentiates the S01234 reporter gene activation induced by *Xwnt8* mRNA *(n* = 2, performed in triplicate, Unpaired *t*-test comparing the DMSO and quercitrin injected groups. ** p < 0.01). Error bars represent mean ± SD. See Figure S2 for the injection scheme.

To validate the quercitrin-mediated Wnt/β-catenin signaling potentiation, we assayed its effect on *Xenopus laevis* early development, a gold standard model to assay Wnt signaling modulation because of its critical relevance to axial patterning [36,45]. Ventral Wnt signaling activation during *Xenopus* early development leads to a secondary axis formation. To investigate if quercitrin potentiates *Xwnt8* (canonical) induced *Xenopus* secondary axis formation (Figure 2), we co-injected 1.6 pmol quercitrin and 1, 3, or 10 pg *Xwnt8* mRNA into the ventral equatorial portion of 4-cell stage *Xenopus* embryos (Figure S2a). Quercitrin enhanced the formation of the *Xwnt8*-induced secondary axis (Figure 2a–g). After 3 pg of *Xwnt8* mRNA injection, 19% of the embryos formed a complete secondary axis, while 40% of the embryos coinjected with 1.6 pmol quercitrin formed a complete secondary axis (Figure 2g). After the injection of 10 pg of *Xwnt8* mRNA, 27% of the embryos formed a complete secondary axis, while 54% of the embryos coinjected with 1.6 pmol quercitrin formed a complete secondary axis (Figure 2g). Additionally, the reporter gene in vivo was assayed by injecting S01234 (*siamois* reporter gene), a Wnt/β-catenin signaling-specific reporter gene [46], into 4-cell *Xenopus* embryos (Figure S2b). Embryos co-injected with quercitrin and *Xwnt8* mRNA had a 2.2-fold activation increase compared to DMSO and *Xwnt8* mRNA-injected embryos (Figure 2h). These data show that quercitrin potentiates the canonical Wnt signaling pathway both in vitro and in vivo.

### 2.2. Quercitrin Facilitates GSK3 S9 Phosphorylation and Hinders GSK3 Activity

**Figure 3:**
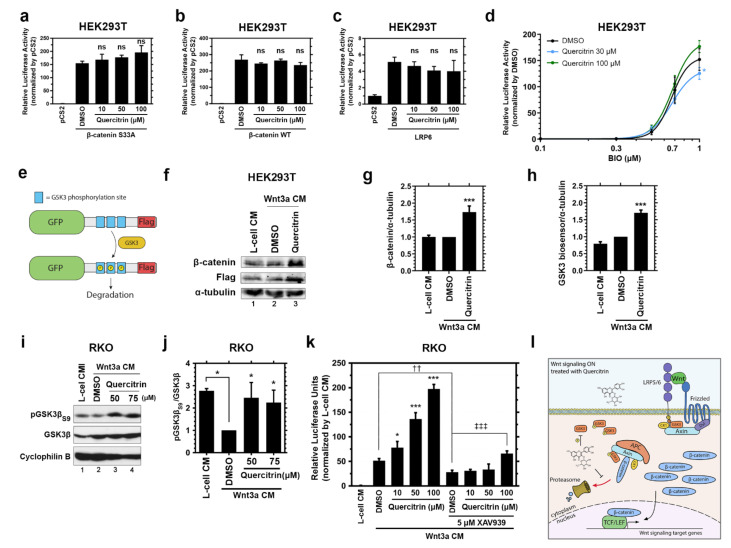
Quercitrin facilitates GSK3 S9 phosphorylation and hinders GSK3 activity. (**a**–**c**) TOPFLASH reporter gene assay of HEK293T cells transfected with (**a**) β-catenin S33A, (**b**) β-catenin WT, or (**c**) LRP6 *(n =* 3, performed in triplicate, one-way ANOVA followed by Dunnett’s multiple comparisons test, ns = not significant). (**d**) Reporter gene assay of HEK293T cells treated with 0.3, 0.5, 0.7, and 1.0 μM of BIO, a GSK3 inhibitor. *(n =* 3, performed in triplicate, Two-way ANOVA followed by Bonferroni’s multiple comparisons test, * p < 0.05). Error bars denote mean ± SD. (**e**) GSK3 biosensor scheme. The biosensor contains the GFP protein at the N-terminus and a FLAG-tag at the C-terminus. The phosphorylation of the biosensor by GSK3 triggers its degradation. (**f**) Immunoblotting assay of HEK293T transfected with the GSK3 biosensor after 6 h treatment. Cells were treated with L-cell CM (lane 1) or Wnt3a CM (lanes 2–5). (**g**) Densitometric analysis of β-catenin/α-tubulin protein levels *(n =* 4, Unpaired *t*-test, two-tailed, *** p < 0.001). Error bars denote mean ± SD. (**h**) Densitometric analysis of GSK3 biosensor/α-tubulin protein levels showing that quercitrin treatment hinders GSK3 activity *(n =* 4, Unpaired *t*-test, two-tailed, *** p < 0.001). Error bars denote mean ± SD. (**i**) Immunoblotting of RKO cells lysate after 24 h treatment. RKO cells were treated with L-cell CM (lane 1) or Wnt3a CM (lanes 2–4). (**j**) Densitometry analysis of pGSK3β_S9_/GSK3β protein levels demonstrating that quercitrin increases the pGSK3β_S9_/GSK3β ratio *(n =* 4, one-way ANOVA followed by Dunnett’s multiple comparisons test, * p < 0.05). Error bars denote mean ± SD. (**k**) Reporter gene assay of RKO B/R cells treated with L-cell CM or Wnt3a CM, quercitrin, and 5 μM XAV939 for 24 h. The treatment reveals that XAV939 impairs the quercitrin potentiation effect *(n =* 3, performed in triplicate, one-way ANOVA followed by Dunnett’s multiple comparisons test considering Wnt3a CM + DMSO condition as control, * p < 0.05, *** *p* < 0.001, or considering Wnt3a CM + DMSO + XAV939 as the control condition, ^‡‡‡^ *p <* 0.001. An Unpaired *t*-test of DMSO and DMSO + XAV939 conditions was performed to assess the XAV939 effect on the reporter gene activity, ^††^ *p* < 0.01). Error bars denote mean ± SD. (**l**) Scheme of quercitrin mechanism of action. Quercitrin hinders GSK3 activity, thus impairing β-catenin degradation and increasing its stabilization. The red arrow indicates β-catenin degradation. The black arrow denotes β-catenin translocation to the nucleus and Wnt target genes transcription.

Once the quercitrin potentiation effect on canonical Wnt signaling was validated, we aimed to solve its mechanism of action. To address this, the signaling was activated at different levels by transfecting HEK293T, a valuable platform, since it can be transfected with high efficiency. We transfected HEK293T cells with the following: β-catenin S33A (constitutively active), β-catenin WT, or LRP6 (Figure 3a–c) and measured the TOPFLASH activity. We also tested whether Quercitrin could potentiate the pathway when HEK293T was co-treated with different concentrations of BIO, a GSK3 inhibitor [47] (Figure 3d). Quercitrin did not potentiate the Wnt signaling when activated with any of these conditions. Additionally, quercitrin had no effect in SW480 cell lines (Figure S3), which harbors an APC mutation [48,49] that disassembles the destruction complex, resulting in increased β-catenin levels.

The LRP6 overexpression inhibits GSK3 (through the PPPSPxS motifs) [50] and disassembles the destruction complex. On the other hand, the activation of Wnt/β-catenin signaling by β-catenin S33A or β-catenin WT overexpression bypass the destruction complex assembly. Considering that quercitrin did not potentiate the Wnt signaling activated either by GSK3 inhibition or by disrupting or bypassing the destruction complex, we hypothesized that the quercitrin mechanism of action relies on the destruction complex physiological dynamic assembly (obtained by close to physiological activation of Wnt/β-catenin signaling), or on GSK3 activity.

To test the quercitrin impact on GSK3 activity, we transfected HEK293T cells with a GSK3 biosensor [51] consisting of a GFP Flag-tagged construct with three GSK3 sites primed by a canonical MAPK phosphorylation site (PXSP). Phosphorylation of the construct by GSK3 marks it for degradation [51] (Figure 3e). HEK293T cells transfected with the GSK3 biosensor were treated with Wnt3a CM at low concentrations together with 50 μM quercitrin for 6 h. Quercitrin treatment led to a 1.6-fold increase in β-catenin and GSK3 (Flag) biosensor levels (Figure 3f–h). The phosphorylation of two sites mainly regulates the activity of the two GSK3 isoforms: S21 and S9, which inhibits GSK3α and GSK3β, respectively, and Y279 and Y216 phosphorylation that increases GSK3α and GSK3β activity, respectively. Since quercitrin increased the GSK3 biosensor levels, suggesting a GSK3 reduced activity, we asked if quercitrin alters the GSK3β inhibitory phosphorylation levels. RKO cells treated with Wnt3a CM and with 50 or 75 μM quercitrin for 24 h showed 2.1-fold increased levels of phosphoGSK3β (pGSK3β) S9 compared to DMSO treated control (Figure 3i,j). Our data show that quercitrin affects GSK3 activity and increases its inhibitory phosphorylation, which results in increased β-catenin stabilization and Wnt/β-catenin signaling activation. We then formulated a mechanism to prevent Wnt signaling activation in a GSK3 reduced activity context. Axin inhibits Wnt/β-catenin in a GSK3-independent manner [52], and Tankyrase (TNKS) activity modulates the Axin levels [53]. Therefore, we hypothesized that the increased Axin levels induced by TNKS inhibition would impair quercitrin-mediated Wnt/β-catenin signaling potentiation. To test this, RKO cells were treated with Wnt3a CM and 10, 50, or 100 μM quercitrin in the presence of 5 μM XAV939, a TNKS inhibitor. XAV939 treatment inhibited the Wnt reporter activity of the Wnt3aCM treated cells by 50% and completely abolished the quercitrin effect at 10 and 50 μM while reducing the quercitrin effect at 100 μM by 67% (Figure 3k). In sum, these data show that quercitrin hinders GSK3 activity leading to increased β-catenin stabilization (Figure 3l).

### 2.3. Quercitrin Potentiates the Canonical Wnt Effects on Hippocampal Synapses In Vitro and Rescues the AβO-Induced Memory Impairment in Mice

**Figure 4:**
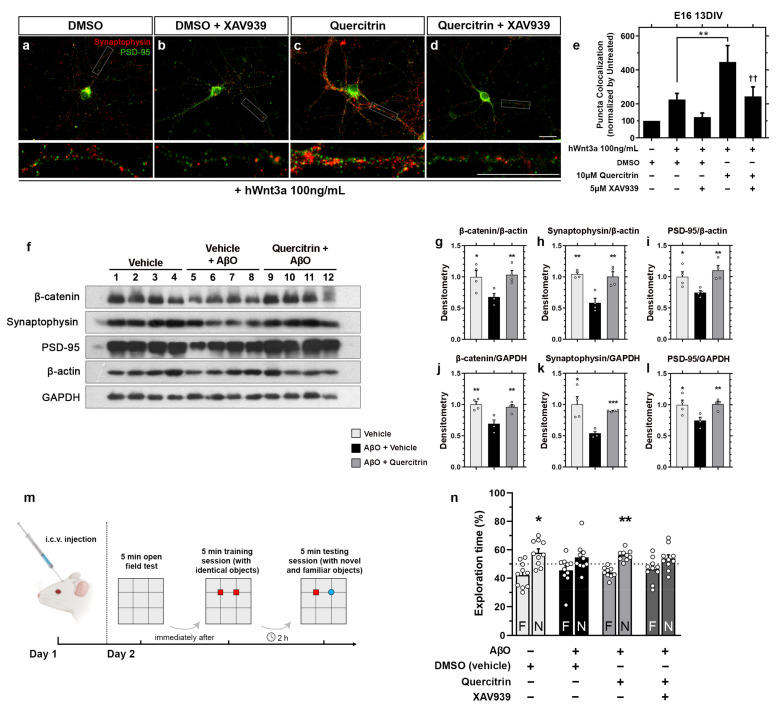
Quercitrin potentiates the canonical Wnt synaptogenic effect and rescues AβO-induced memory impairment in mice. (**a**–**d**) Synaptophysin and PSD-95 immunocytochemistry of E16 13DIV cultured hippocampal neurons treated with XAV939 and/or quercitrin for 24 h. Scale bars: 20 μm. (**e**) Synaptophysin/PSD-95 colocalized puncta quantification of (**a**–**d**), showing that quercitrin potentiates the recombinant Wnt-mediated synaptogenesis in vitro, which is impaired by XAV939 *(n =* 3, performed in duplicate, one-way ANOVA statistical analysis followed by Newman–Keuls multiple comparisons test. ** p < 0.01, comparing hWnt3a + DMSO, and hWnt3a + quercitrin conditions. ^††^ *p <* 0.01, comparing hWnt3a + quercitrin and hWnt3a + quercitrin + XAV939 conditions). Scale bars denote 20 μm. (**f**) Immunoblotting analysis of injected mice hippocampus lysate staining for β-catenin, synaptophysin, and PSD-95. β-actin was used as a cytoskeleton loading control. GAPDH was used as a metabolic loading control. Each lane represents a different animal. The animals were injected with vehicle (lanes 1–4), vehicle + AβO (lanes 5–8), or quercitrin + AβO (lanes 9–12). (**g**–**l**) Densitometric quantification of (**f**) showing that quercitrin rescues the decreased β-catenin, synaptophysin, and PSD-95 protein levels caused by AβO ICV injection *(n =* 4, Unpaired *t*-test, two-tailed, considering the AβO + vehicle as the control condition, * p < 0.05, ** p < 0.01, *** p < 0.001). Error bars denote mean ± SEM. Each white circle denotes one animal. (**m**) Workflow scheme of the i.c.v. injections, object recognition training, and testing. Red square represents a familiar object, and blue object represents a novel object. (**n**) Novel object recognition (NOR) test, 24 h after i.c.v. injection. Quercitrin reverses the memory impairment caused by AβO injection, which is impaired by XAV939 *(n =* 10, One sample *t*-test, theoretical mean value of 50%, * p < 0.05, ** p < 0.01). F: familiar object, N: novel object. Error bar represents mean ± SEM. See also Figure S5 for NOR training and open field arena test data.

In vivo and in vitro studies demonstrated that chronic treatment with extracts enriched with polyphenols or purified flavonoids induces cognitive and behavioral improvements, mainly associated with an increase in the number of synapses [27,54]. Since canonical Wnt signaling activation has a role in synaptogenesis [55] and quercitrin potentiates the signaling activation, we asked whether it would enhance synaptogenesis. To address this question, we isolated and cultured E16 hippocampal mouse neurons for 13 days in vitro (DIV). The number of synapses was analyzed by quantifying the colocalization puncta of the pre- and postsynaptic markers, synaptophysin, and (Postsynaptic Density Protein-95) PSD-95, respectively. The treatment of neuronal cultures with recombinant hWnt3a induced a 2-fold increase in the colocalized puncta count (*p* < 0.001, Welch *t*-test statistical analysis). The addition of 10 μM quercitrin simultaneously to hWnt3a enhanced the hWnt3a effect on synapse number by 2-fold (4.5-fold increase when compared to the untreated condition) (Figure 4a–e). The addition of 5 μM XAV939 impaired the synaptogenic effect of hWnt3a either in the presence of quercitrin (Figure 4e). Treatment of neuronal cultures with 10 μM quercitrin alone did not alter the number of synaptophysin/PSD-95 colocalized puncta (Figure S4). These data show that quercitrin potentiates the synaptogenic effect of canonical Wnt signaling and that a downstream inhibition can impair this effect.

Synapse loss is an essential hallmark of several memory dysfunctions [56]. We thus investigated whether canonical Wnt potentiation induced by quercitrin had an effect on synaptic protein amount. We performed immunoblotting with the hippocampal lysate of AβO-infused mice to assess the amount of β-catenin, synaptophysin, and PSD-95 protein levels (Figure 4f). The densitometric analysis was normalized by the following two loading controls: β-actin, a cytoskeleton protein, and GAPDH, a metabolic protein (Figure 4g–i). AβO injection reduced synaptophysin, PSD-95, and β-catenin protein levels, which were rescued by quercitrin injection (Figure 4j–l). The β-catenin protein levels suggest that AβO injection resulted in an inhibited Wnt signaling scenario, which was rescued by quercitrin injection.

Our finding that quercitrin presents synaptogenic potential in vitro and prevents AβO-induced synapse loss prompted us to examine its impact on memory dysfunction triggered by the oligomers (Figure 4m). As expected, vehicle-infused mice learned the novel object recognition (NOR) memory task, as demonstrated by a longer exploration of the novel object over the familiar one (Figure 4n). In contrast, AβO-infused mice failed the NOR task, and quercitrin treatment rescued cognitive impairment induced by AβO (Figure 4n). Co-treatment with XAV939 abolished quercitrin’s effect on memory dysfunction caused by AβO, suggesting that the effect of this flavonol relies on canonical Wnt signaling activation. The injection of vehicle, quercitrin, XAV939, or AβO did neither affect the exploratory function both in the training or test sessions of the NOR task (Figure S5).

In summary, quercitrin rescues synaptic damage and memory impairment induced by AβO in mice. Furthermore, the following evidence correlates the quercitrin effect with Wnt/β-catenin signaling modulation: (i) the β-catenin levels are simultaneously modulated with the memory impairment rescue, and (ii) the downstream inhibition by XAV939 abrogates quercitrin beneficial effect.

## 3. Discussion

The Wnt signaling origin coincided with metazoa and was crucial for patterning axes during embryonic development [57]. Among vertebrates, besides its axis patterning function, Wnt signaling has a relevant role in regulating dendritogenesis, axon guidance, and synaptogenesis [58]. Although not entirely solved, the Wnt signaling role in the mature CNS persists throughout adulthood. The Wnt ligands are still expressed in the adult brain [59] and act in synapse maintenance and plasticity [60]. Furthermore, Wnt signaling is often downregulated in neurodegenerative diseases, such as AD [61]. In this regard, Wnt signaling activation or potentiation reverts cognitive impairment in mouse models [16]. Intriguingly, the description of novel Wnt signaling activating compounds is less frequent than the inhibitors [62]. This disparity is even broader when compounds in the clinical trial phase are compared, in which there are three times more inhibiting than activating compounds [62].

Here, we describe quercitrin, a flavonol first characterized by the Austrian chemist Heinrich Hlasiwetz [63,64], abundantly found in buckwheat and several oak species, as a novel Wnt signaling potentiator molecule that cannot activate the pathway in the absence of co-treatment with Wnt3a CM or recombinant Wnt3a (Figure 1a,b). The potentiation effect is already noticeable after 4 h of treatment (Figure 1e), suggesting a direct effect on β-catenin protein stabilization. Accordingly, the Wnt signaling gene-reporter activity (B/R) is potentiated after 6 h of treatment with quercitrin (Figure 1c,d). Suggesting that the quercitrin potentiation effect has a quick transcriptional effect on Wnt signaling. This potentiation effect enhances the kinetic of β-catenin stabilization, increases pLRP6 levels, and decreases pβ-catenin levels (Figure 1g). The Wnt signaling potentiation was validated using *Xenopus laevis* development, in which quercitrin increased the occurrence of the *Xwnt8*-induced secondary axis (Figure 2g), while also enhancing the Wnt signaling specific reporter gene (S01234) activity (Figure 2h). The canonical Wnt signaling potentiation was assayed by measuring the occurrence of the *Xwnt8* induced secondary axis phenotype inspired by the previous description of QS11, a Wnt signaling synergist small molecule [65].

We addressed the quercitrin mechanism of action by performing an epistasis assay in vitro. Our data showed that quercitrin increases the phosphorylated serine 9 GSK3β levels (Figure 3i,j). N-terminal phosphorylation of GSK3 results in self-recognition and, therefore, blocks its kinase activity [66]. We validated the GSK3 decreased activity after quercitrin treatment by using a GSK3 activity biosensor (Figure 3e–h) [51]. Other compounds, such as BIO and andrographolide, modulate the Wnt signaling by altering GSK3 N-terminal phosphorylation [67,68]. The modulation of Axin levels impairs Wnt signaling despite GSK3 activity, since the Axin mutant lacking the GSK3 binding region modulates the Wnt signaling pathway [52]. Corroborating the GSK3 mediated mechanism, XAV939 treatment [53], a TNKS inhibitor, effectively impaired the quercitrin potentiation effect. Curiously, quercitrin impairs GSK3 activity and increases its N-terminal phosphorylation level; however, it only potentiates the pathway upon Wnt signaling upstream stimulation (Figure 3l). This puzzle suggests a more complex mechanism in which quercitrin leads to an increase in phosphorylated GSK3 levels.

Because of the Wnt signaling role in neurodegeneration, we envisioned assaying the quercitrin potentiation in this context [15,16]. We showed that quercitrin potentiates the recombinant Wnt3a synaptogenic effect, which can be blocked by the XAV939 treatment (Figure 4a–e). Next, we intended to assess the Wnt signaling potentiation effect in vivo via i.c.v. injection [69,70]. Opportunely, the Wnt signaling potentiator WASP-1 displays in vivo beneficial effects, without the need for supplemental Wnt signaling activation, probably because of endogenous Wnt signaling [16,71]. Thus, we demonstrated that the quercitrin injection reverts the reduction in both the pre- and postsynaptic protein levels induced by AβO i.c.v. injection (Figure 4f–l). The levels of the synaptic proteins PSD-95 and synaptophysin, known to be regulated by the canonical Wnt pathway [72], fluctuated accordingly to the β-catenin protein levels, suggesting a quercitrin-mediated Wnt signaling activation (Figure 4f–l). Finally, quercitrin rescued AβO-mediated memory impairment, which was blocked by XAV939 co-injection (Figure 4m,n).

These data corroborate the hypothesis that activation or potentiation of Wnt signaling can revert memory impairment, typical of Alzheimer’s disease models [7,8,17,73,74,75]. One reason for this effect might be the modulation of pre- and postsynaptic components upon Wnt signaling activation [72]. Also, the Wnt function of regulating the hippocampal neurons LTP may play a significant role in this effect [5]. Positive Wnt signaling modulators might also promote neurogenesis by stimulating the proliferation of brain stem cells [59,76,77].

A pivotal protein that connects Wnt signaling and Alzheimer’s disease is GSK3β. A Tet/GSK3β transgenic mouse that overexpresses GSK3β in the adult brain displays hyperphosphorylated tau, neurodegeneration, and spatial learning deficit, resembling AD [78,79]. The GSK3β active form colocalizes with abnormally hyperphosphorylated tau in human brain tissue [80]. The Wnt signaling activation hinders GSK3β activity by phosphorylated LRP5/6 proteins [50,81,82]. For this reason, Wnt signaling activation by canonical Wnt ligands impairs apoptosis, reduces tau hyperphosphorylation, and prevents neurite loss in primary hippocampal cultures treated with β-amyloid [17,83]. Here, we speculate that quercitrin rescues memory impairment caused by AβO injection by decreasing GSK3 activity and, therefore, increasing the pre- and postsynaptic proteins levels.

It is worth noting that Wnt signaling is aberrantly active in a myriad of cancers [2] and many natural compounds that inhibit Wnt signaling also impair tumor growth [39,84,85,86,87,88]. However, an activating compound could further fuel tumorigenesis [89]. Fortunately, our quercitrin epistasis assay showed that quercitrin does not potentiate the Wnt pathway triggered by either β-catenin, LRP6, or S33A β-catenin overexpression (Figure 3a–c). Similarly, quercitrin does not potentiate the pathway in SW480 cell lines (Figure S3), a colorectal cancer cell line harboring an APC mutation that leads to Wnt signaling overactivation [48,49], suggesting that quercitrin might not contribute to tumor formation. However, depending on the scenario, quercitrin might potentiate the signaling, for example, if the abundance of the Wnt ligands or the lack of the secreted Wnt ligand inhibitors drove the aberrant activation.

The compounds that positively modulate the canonical Wnt pathway have a wide range of applications in situations where the signaling is downregulated, besides displaying a therapeutic potential in neurodegeneration. Some examples of these applications are in the treatment of hair growth [90,91], skin pigmentation disorders [92], wound healing [93], non-syndromic cleft lip [94,95], transient cerebral ischemia [96], chronic obstructive pulmonary disease and emphysema [97], and bone diseases [98]. In fact, previous works showed that quercitrin treatment reduces osteoclast activity in vitro and in vivo [99], stimulates osteoblast differentiation [34], inhibits adipocyte differentiation [100], and has beneficial effects in diarrhea models, probably by stimulating intestinal epithelium regeneration [101,102]. All of these effects correlate to Wnt signaling activation or potentiation [103,104], corroborating our findings.

We previously demonstrated the effect of different flavonoids on central and peripheric neural progenitor differentiation and survival [105,106,107]. These and several other works suggest the impact of flavonoids on neuronal physiology and implicate these molecules in the control of neurotransmission, synapse formation, plasticity, neurogenesis, and cognitive improvements in several animal models [54,108,109]. Moreover, in humans, the long-term consumption of polyphenol-rich diets has been associated with better cognitive performance due to an enhanced function of the hippocampal dentate gyrus, as well as slower rates of cognitive decline in older adults [110,111]. These data are corroborated by the fact that many flavonoids can cross the blood-brain barrier and modulate brain function [54]. In agreement, the administration of flavonoids or polyphenol-rich extracts is linked to learning and memory improvements in aged animals, including in preclinical models for neurodegenerative diseases [25,112,113].

A growing number of studies have elucidated the cellular targets and molecular mechanisms of flavonoid actions in the CNS, including neuronal-binding sites and downstream signaling pathways modulated by these compounds [25,114]. We recently demonstrated that the flavonoid hesperidin improved memory performance in healthy adult mice due to increased hippocampal synapse formation and activation of the TGF-β1 pathway [27].

Quercitrin is a quercetin O-glycoside, i.e., a quercetin substituted by an α -L-rhamnosyl moiety at position C-3. Intriguingly, quercetin is a Wnt signaling inhibitor [36,37], as well as isoquercitrin [39], a quercetin with a β-d-glucosyl residue attached at position C-3. Rutin, a quercetin with glucose and rhamnose sugar groups attached at position C-3, does not modulate the signaling [36]. These works suggest that sugar moieties affect the quercetin activity and might help unravel novel pharmacophores.

In conclusion, here we describe a novel function for quercitrin. By employing in vitro and in vivo gold standard Wnt/β-catenin assays, we defined quercitrin as a novel Wnt signaling potentiator. We combined reporter gene assays and the activation of the canonical Wnt pathway at different levels to show that quercitrin hinders GSK3 activity. Finally, our data show a potential therapeutic application for quercitrin in memory deficit and evidence of canonical Wnt signaling role in neurodegeneration. Our results also help to explain previously described quercitrin effects and facilitate the quest for pharmacophores that potentiate the Wnt signaling. Further experiments should be performed to provide a better understanding of the quercitrin mechanism on Wnt signaling, and its potential effect on AD treatment.

## 4. Materials and Methods

### 4.1. Reagents and Cell Culture

HEK293T was purchased from ATCC, and RKO pBAR/Renilla (B/R), and SW480 B/R cell lines were kindly donated by Professor Xi He (Boston Children’s Hospital—Harvard Medical School). Every cell line was cultured with DMEM-F12 (Gibco, Waltham, MA, USA) supplemented with 10% FBS (Gibco). The flavonoid quercitrin (Sigma, Burlington, MA, USA, #00740580—CAS Number 522–12-3) was solubilized into DMSO (Sigma, Burlington, MA, USA) at 50 mM and stored at −20 °C. XAV939 (Sigma, Burlington, MA, USA) was solubilized into DMSO at 10 mM and stored at −20 °C. BIO was purchased from Sigma (#B1686—CAS Number 667463–62-9) and solubilized into DMSO (Sigma, Burlington, MA, USA) at 10 mM. L-cell and L-Wnt3a cell lines were obtained from ATCC, and the conditioned medium was obtained according to ATCC protocol. pCS2 GFP-GSK3-MAPK was a gift from Professor Edward De Robertis, University of California, Los Angeles (Addgene plasmid #29689; http://n2t.net/addgene:29689 (accessed on 9 September 2022); RRID: Addgene_29689) [51]. For testing quercitrin effect, the vehicle DMSO was considered as the control group. The absence of Wnt3a CM treatment or the treatment with XAV939 was considered as the negative control group. The addition of Wnt3a CM or rhWnt3a was considered as the positive control group.

### 4.2. Xenopus Laevis Embryo Manipulation

Adult frogs (Nasco Inc., Fort Atkinson, WI, USA) were stimulated with 1000 IU human chorionic gonadotropin (Ferring Pharmaceuticals, Kiel, Germany) according to the Animal Care and Use Ethics Committee from the Federal University of Rio de Janeiro and were approved by this committee under permission number 152/13. *Xenopus* embryos were obtained by in vitro fertilization and staged according to Nieuwkoop and Farber [115]. *Xwnt8* mRNA was synthesized by linearizing *Xwnt8*-containing plasmid with NotI and transcribing the mRNA using SP6 RNA polymerase (mMESSAGE, mMACHINE SP6 Transcription kit, Invitrogen, Waltham, MA, USA). For canonical Wnt signaling reporter gene assay, two 4-cell stage embryos transverse blastomeres were injected with 4 nL containing 280 pg of S01234-luciferase plasmid, 50 pg of Tk-*Renilla* plasmid, 10 pg of *Xwnt8* mRNA, and 0.8 pmol of quercitrin or DMSO each, for a total of 1.6 pmol of quercitrin per embryo. For inducing a secondary axis, two 4-cell stage ventral blastomeres were ventrally injected around the equatorial region with 1, 3, or 10 pg of *Xwnt8* mRNA per embryo together with 1.6 pmol quercitrin or DMSO. We determined the quercitrin injection concentration based on other compounds [45,87]. Following microinjections, embryos were cultivated in 0.1× Barth (8.89 mM NaCl; 0.1 mM KCl; 0.24 mM NaHCO_3_; 0.08 mM MgSO_4_·7H_2_O; 1 mM Hepes; 0.03 mM Ca(NO3)2.4H2O; 0.04 mM CaCl_2_·2H_2_O; pH 7.7) until control embryos reached stage 10. Triplicates of four embryos were lysed using PLB (Promega, Madison, WI, USA) and assayed for Firefly and *Renilla* luciferase activity using the Dual-Luciferase Reporter Assay. All experiments were performed at 22 °C and in triplicate. *Xwnt8* mRNA-injected embryos were considered a positive control group, while uninjected embryos were considered the negative control group.

### 4.3. Mature Hippocampal Neuronal Culture

Primary hippocampal neuronal cultures were performed according to [116] using E16 Swiss mice and assayed after 12 days in vitro. Briefly, cultures were prepared and maintained in Neurobasal medium supplemented with B-27, penicillin, streptomycin, fungizone, L-glutamine, and cytosine arabinoside (0.65 μM, Sigma, Burlington, MA, USA) at 37 °C in a humidified 5% CO_2_, 95% air atmosphere for 12 days in vitro. After, cultures were cotreated for 24 h with 100 ng/mL recombinant hWnt3a plus DMSO or 10 μM quercitrin. To inhibit the Tankyrase (TNKS) activity and therefore suppress Wnt signaling, neuronal cultures were cotreated with 100 ng/mL recombinant hWnt3a plus DMSO or 10 μM quercitrin in the presence of 5 μM XAV939 for 24 h. The experiment was performed in triplicate.

### 4.4. Mice Experiments

Sixteen embryonic days (E16) Swiss mice were used for neuronal cultures. For rodent in vivo experiments, three-month-old male Swiss mice from our animal facilities, housed in groups of five per cage with free access to food and water, under a 12 h light/dark cycle, controlled temperature, and humidity were used. All procedures followed the “Principles of Laboratory Animal Care” (US National Institutes of Health) and were approved by our local Animal Care and Use Committee (Federal University of Rio de Janeiro, Health Sciences Center, protocol #106/19, and #006/18). Experiments were performed according to Brazilian Guidelines on Care and Use of Animals for Scientific and Teaching Purposes (DBCA).

### 4.5. AβO Infusion and Pharmacological Treatments

For infusion into the lateral ventricle (i.c.v.), animals were anesthetized with 2.5% isoflurane (Cristália; São Paulo, Brazil) using a vaporizer system (Norwell, MA, USA) and were gently restrained briefly during the injection procedure. A 2.5 mm-long 30 G needle was unilaterally inserted approximately 1 mm to the midline point equidistant from each eye and parallel to a line drawn through the anterior base of the eye as previously described. Using a Hamilton syringe, solutions (3 µL) containing vehicle (0.53% DMSO in phosphate-buffered saline solution [PBS]), recombinant Wnt3a (100 ng), quercitrin (300 pmol), XAV939 (100 pmol) or combined quercitrin/XAV939 (300 pmol quercitrin, 100 pmol XAV939) were slowly infused. After one hour, mice were again anesthetized and received a second injection (3 µL) with vehicle (PBS) or AβOs (10 pmol) at the same place. Mice showing any signs of misplaced injections or brain hemorrhage (~5% of animals throughout our study) were excluded from further analysis.

### 4.6. Behavioral Analysis

The behavioral evaluation was performed on the following day of pharmacological treatment. Mice were placed in the experimental room under controlled light (indirect fluorescent light) and temperature (23 °C) conditions for at least one hour before analysis. First, mice were submitted to a five min-long open-field arena test (habituation phase), in which they were placed at the center of an arena (30 cm × 30 cm × 45 cm) divided into nine equal quadrants by imaginary lines on the floor and allowed to explore it for 5 min freely. Total locomotor activity and time spent at the center or the periphery of the arena were recorded using ANY-maze software (v7.1, Stoelting Company, Wood Dale, IL, USA). The arena was thoroughly cleaned with 70% ethanol in between trials to eliminate olfactory cues. Immediately after habituation, mice were submitted to the novel object recognition test. During a 5-min long training session, animals were placed at the center of the same arena, now in the presence of two identical objects. During sessions, objects were fixed to the arena’s floor using tape to prevent displacement caused by the exploratory activity of the animals. A trained researcher recorded the amount of time mice spent exploring each object. Sniffing and touching the object were considered exploratory behavior. The arena and objects were cleaned thoroughly with 70% ethanol in between trials to eliminate olfactory cues. Two hours after training, animals were again placed in the arena for the test session, with one of the objects used in the training session having been replaced by a new one. Again, the amount of time spent exploring familiar and novel objects was measured. Test objects were made of plastic and had different shapes and colors. Preliminary tests showed that none of the objects used evoked innate preference. Results were expressed as a percentage of time exploring each object during the training or test sessions or as total exploration during each session. Data were analyzed using a one-sample Student’s *t*-test comparing the mean exploration percentage time for each object with the chance value of 50%. Animals that recognize the familiar object as such (i.e., learn the task) explore the novel object > 50% of the total time. For the NOR test, the AβO + DMSO group was considered the positive control group. The DMSO-only injected group was considered the negative control group.

### 4.7. Dual-Luciferase Assay

HEK293T (1.5 × 10^4^) cells were seeded into 96-well plates and transfected using Lipofectamine 3000 (Invitrogen, Waltham, MA, USA) with TOPFLASH (100 ng/well) and *Tk-Renilla* (50 ng/well) according to manufacturer protocol. RKO B/R (1.5 × 10^4^) cells were seeded into 96-well plates. Cells were treated with control media, L-cell conditioned medium (CM), Wnt3a CM, recombinant hWnt3a (StemRD, Burlingame, CA, USA, W3A-H-005), or other specified conditions. After treatment, cells were lysed with Passive Lysis Buffer (PLB—Promega, Madison, WI, USA) and assayed for Firefly and *Renilla* luciferase activity using the Dual-Luciferase Reporter Assay (Promega, Madison, WI, USA, #E1960). Luminescence was measured using the Modulus II^TM^ Microplate Multimode Reader (Promega, Madison, WI, USA). Every assay was repeated at least three times and in triplicate.

### 4.8. Immunoblotting

HEK293T or RKO cells were lysed using RIPA (50 mM Tris-HCl 50, 150 mM NaCl, 1% IGEPAL CA-630, 0,5% Sodium Deoxycholate and 0.1% SDS, pH 7.4) or Triton (50 mM Tris, 150 mM NaCl, 1 mM EDTA, 1% Triton X-100, 10% Glycerol, pH 7.5) lysis buffer and phosphatase/protease inhibitors (Pierce™ Protease and Phosphatase Inhibitor Mini Tablets, Waltham, MA, USA, #A32959). Cell lysates were mixed with Laemmli buffer (2% SDS, 10% Glycerol, 5% 2-mercaptoethanol, 0.002% bromophenol blue, and 125 mM Tris HCl, pH 6.8) and heated at 95 °C for 5 min. Protein samples were separated on the SDS-PAGE gel and transferred to the Immobilon-E membrane (Millipore, Burlington, MA, USA). Membranes were blocked for 1 h or overnight with TBS-T 2% PVP (Polyvinylpyrrolidone, Sigma, Burlington, MA, USA, #PVP40). Indirect immunochemistry using horseradish peroxidase-conjugated secondary antibodies (Invitrogen, Waltham, MA, USA, #31460 and #31430) was detected using SuperSignal West Pico chemiluminescent substrate (Pierce, Waltham, MA, USA) or SuperSignal Femto Maximum Sensitivity Substrate (Pierce, Waltham, MA, USA) and exposed in Kodak X-OMAT film. The immunoblot of the GSK3 was transferred to an Immobilon-FL membrane (Millipore, Burlington, MA, USA), blocked with the Blocking Buffer (LI-COR, Lincoln, NE, USA, 927–70001), and incubated with the IRDye 680 (1:15,000, LI-COR, Lincoln, NE, USA, 926-68072), IRDye 800 (1:15,000, LI-COR, Lincoln, NE, USA, 926-32213) secondary antibodies and exposed in the Odyssey Imaging System (LI-COR, Lincoln, NE, USA).

The following primary antibodies were used according to manufactures concentration: α-tubulin (1:2000, Sigma, Burlington, MA, USA, #T9026), β-actin (1:5000, SCBT, Dallas, TX, USA, #sc-47778), β-catenin (1:2000, BD, East Rutherford, NJ, USA, #610154), phosphorylated β-catenin S33, S37 (1:500, CST, Danvers, MA, USA, #2009), Cyclophilin B (1:3000, CST, Danvers, MA, USA, # SAB4200201), Flag-M2 (1:2000, Sigma, Burlington, MA, USA, #F1804), GAPDH (1:5000, CST, Danvers, MA, USA, #5174), GSK3β (1:2000, CST, Danvers, MA, USA, #9315), phosphorylated GSK3β S9 (1:1000, CST, Danvers, MA, USA, #9323), LRP6 (1:1000, CST, Danvers, MA, USA, #C47E12), phosphorylated LRP6 S1490 (1:1000, CST, Danvers, MA, USA, 2568), synaptophysin (1:1000, Millipore, Burlington, MA, USA, #MAB368) and PSD-95 (1:1000, Abcam, Cambridge, UK, #ab18258). Densitometric analyses of the immunoblots were performed using NIH ImageJ software, Bethesda, MD, USA (FIJI version 1.53c).

### 4.9. Immunocytochemistry and Synaptic Puncta Analysis

Immunocytochemistry and synaptic puncta analysis were performed according to [27]. Briefly, cultured neurons were fixed with 4% PFA for 15 min, washed with PBS, and incubated with a blocking solution, composed of 3% bovine serum albumin, 5% normal goat serum (Sigma, Burlington, MA, USA), and 0.2% Triton X-100 in PBS for 1 h. Then, the cells were incubated overnight at 4 °C with the following primary antibodies diluted in a blocking solution: mouse anti-synaptophysin (1:1000; Millipore, Burlington, MA, USA, #MAB368) and rabbit anti-PSD-95 (1:100; CST, Danvers, MA, USA, #2507). After, the cells were extensively washed with PBS and incubated for 2 h at room temperature with the following secondary antibodies: AlexaFluor 546-conjugated goat anti-mouse IgG (1:1000; Invitrogen, Waltham, MA, USA, #A-11003), or AlexaFluor 488-conjugated goat anti-rabbit IgG (1:300; Invitrogen, Waltham, MA, USA, #A-11008). Nuclei were counterstained with DAPI (Sigma, Burlington, MA, USA), and micrographs were acquired in a TE 2000 Nikon microscope.

Hippocampal neurons were randomly selected based on DAPI staining, and 10–20 fields were imaged per experimental condition. The green and red channels were merged and quantified using the Puncta Analyzer plug-in in NIH ImageJ. The experiments were performed in duplicate, and each result represents the mean value of three independent hippocampal neuronal cultures.

### 4.10. Statistical Analysis

Comparison among treatments was performed employing the Student *t*-test or one-way analysis of the variance (ANOVA) and Dunnett post hoc comparison test unless declared otherwise. Statistical significance was defined as * p < 0.05, ** p < 0.01 or *** p < 0.001, unless specified otherwise, and ns stands for not significant. DMSO-treated conditions were considered as the control group for the statistical analyses unless stated otherwise. Every experiment was repeated at least three times unless specified otherwise. Behavioral experiments were performed with ten mice for each condition. Every graph displays the mean ± SD or mean ± SEM, depicted in every figure legend. The statistical analyses were performed using GraphPad Prism version 7.00 for Windows, GraphPad Software, La Jolla, CA, USA, www.graphpad.com.

## Data Availability

Not applicable.

## Supplementary Materials

The following supporting information can be downloaded at: https://www.mdpi.com/article/10.3390/ijms232012078/s1.
