# Supplementary material for: The Flavonol Quercitrin Hinders GSK3 Activity and Potentiates the Wnt/β-Catenin Signaling Pathway
Source: Int J Mol Sci. 2022 Oct 11;23(20):12078. doi: 10.3390/ijms232012078 (PMC9602613; doi:10.3390/ijms232012078)
Supplement: Supplementary file 1 [file ijms-23-12078-s001.zip › ijms-1882659-supplementary.pdf]

Supporting Information for

The flavonol quercitrin hinders GSK3 activity and potentiates the Wnt/β-catenin signaling pathway

Danilo Predes[1,†], Lorena A. Maia[1], Isadora Matias[1], Hannah Paola Mota Araujo[2], Carolina Soares[2], Fernanda G. Q. Barros-Aragão[2], Luiz F. S. Oliveira[1], Renata R. Reis[1], Nathalia G. Amado[1,‡], Alessandro B. C. Simas[3], Fabio A. Mendes[1], Flávia C. A. Gomes[1], Claudia P. Figueiredo[2], Jose G. Abreu[1]

[1] Instituto de Ciências Biomédicas, Universidade Federal do Rio de Janeiro, Rio de Janeiro 21941-902, Brazil

[2] Faculdade de Farmácia, Universidade Federal do Rio de Janeiro, Rio de Janeiro 21941-901, Brazil

[3] Instituto de Pesquisas de Produtos Naturais Walter Mors, Universidade Federal do Rio de Janeiro, Rio de Janeiro 21941-901, Brazil

* Correspondence: garciajr@icb.ufrj.br; Tel: +55-21-3938-6486

† Current Address: F. M. Kirby Neurobiology Center, Boston Children's Hospital, Harvard Medical School, Boston, MA 02115, USA

‡ Current Address: Department of Urology, University of Texas Southwestern Medical Center, Dallas, TX 75390, USA.

This PDF file includes:

Materials and Methods
Figures. S1 to S5

Materials and Methods

KEY RESOURCES TABLE

REAGENT or RESOURCE	SOURCE	IDENTIFIER
Antibodies		
Synaptophysin	Vector Laboratories	Cat# VP-S285, RRID:AB_2336747
Synaptophysin	Millipore	Cat# MAB368, RRID:AB_94947
Homer-1	Abcam	Cat# ab184955, RRID:AB_2744679
PSD-95	Cell Signaling Technology	Cat# 2507, RRID:AB_561221
PSD-95	Abcam	Cat# ab18258, RRID:AB_444362
α-tubulin	Sigma	Cat# T9026, RRID:AB_477593
β-actin	SCBT	Cat# sc-47778 HRP, RRID:AB_2714189
β-catenin	BD	Cat# 610154, RRID:AB_397555
phosphorylated β-catenin S33, S37	Cell Signaling Technology	Cat# 2009, RRID:AB_2088238
Cyclophilin B	Cell Signaling Technology	Cat# SAB4200201, RRID:AB_10743624
Flag-M2	Sigma	Cat# F1804, RRID:AB_262044
GAPDH	Cell Signaling Technology	#Cat# 5174, RRID:AB_10622025
GSK3β	Cell Signaling Technology	Cat# 9315, RRID:AB_490890
phosphorylated GSK3β S9	Cell Signaling Technology	Cat# 9323, RRID:AB_2115201
LRP6	Millipore	Cat# MAB368, RRID:AB_94947
AlexaFluor 488 Goat anti-Rabbit	Invitrogen	Cat# A-11008, RRID:AB_143165
AlexaFluor 488 Goat anti-Mouse	Invitrogen	Cat# A-11001, RRID:AB_2534069
AlexaFluor 546 Goat anti-Mouse	Invitrogen	Cat# A-11003, RRID:AB_141370
AlexaFluor 555 Goat anti-Rabbit	Invitrogen	Cat# A-21428, RRID:AB_141784
IRDye 680	LI-COR	Cat# 926-68072, RRID:AB_10953628
IRDye 800	LI-COR	Cat# 926-32213, RRID:AB_621848
Goat anti-Rabbit IgG, HRP	Invitrogen	Cat# 31460, RRID:AB_228341
Goat anti-Mouse IgG, HRP	Invitrogen	Cat# 31430, RRID:AB_228307

Chemicals, Peptides, and Recombinant Proteins		
Quercitrin	Sigma	Cat# 00740580, CAS Number 522-12-3
DMSO	Sigma	Cat# D4540,<br>CAS Number 67-68-5
XAV939	Sigma	Cat# X3004, CAS Number 284028-89-3
BIO	Sigma	Cat# B1686, CAS Number 667463-62-9
Cytosine arabinoside	Sigma	Cat# C1768, CAS Number 147-94-4
rhWnt3a	StemRD	Cat# W3A-H-005
Pierce™ Protease and Phosphatase Inhibitor Mini Tablets	Thermo Scientific	Cat# A32959
Prolong Gold Antifade	Invitrogen	Cat# P10144
Lipofectamine 3000	Invitrogen	Cat# L3000015
Lithium chloride (LiCl)	Sigma	Cat# L4408
Immobilon-E	Millipore	Cat# IEVH85R
Immobilon-FL	Millipore	Cat# IPFL00010
SuperSignal West Pico	Pierce	Cat# 34079
SuperSignal Femto Maximum Sensitivity Substrate	Pierce	Cat# 34094
Blocking Buffer	LI-COR	926-32213
Critical Commercial Assays		
Dual-Luciferase Reporter Assay System	Promega	Cat# E1960
mMESSAGE mMACHINE SP6 Transcription kit	Invitrogen	Cat# AM1340
Experimental Models: Cell Lines		
HEK293T	ATCC	RRID:CVCL_0063
RKO B/R	(Major *et al*, 2007)	N/A
SW480 B/R	(Predes *et al*, 2019)	N/A
L-cell	ATCC	RRID:CVCL_4536
L-Wnt3a	ATCC	RRID:CVCL_0635
Experimental Models: Organisms/Strains		
Xenopus laevis	Xenopus Express, Inc	N/A
Swiss mice	Federal University of Rio de Janeiro	N/A
Recombinant DNA		
pCS2	ATCC	RRID:Addgene_16331
pCS2 GFP-GSK3-MAPK	ATCC	RRID: Addgene_29689
TOPFLASH	ATCC	RRID:Addgene_12456
Tk-*Renilla*	Promega	Cat# E2241
pCS2 *Xwnt8*	ATCC	RRID:Addgene_16865

S01234	(Brannon *et al*, 1997)	N/A
pCS2 *LRP6*	ATCC	RRID:Addgene_27242
Wnt3a	ATCC	RRID:Addgene_43810
Software and Algorithms		
ImageJ	NIH	https://imagej.nih.gov/ij/
Puncta Analyzer	ImageJ 1.29 NIH	RRID: SCR_003070
Prism 7	GraphPad	https://www.graphpad.com/scientific-software/prism/
ANY-maze software	Stoelting Company	http://www.anymaze.co.uk/
Other		

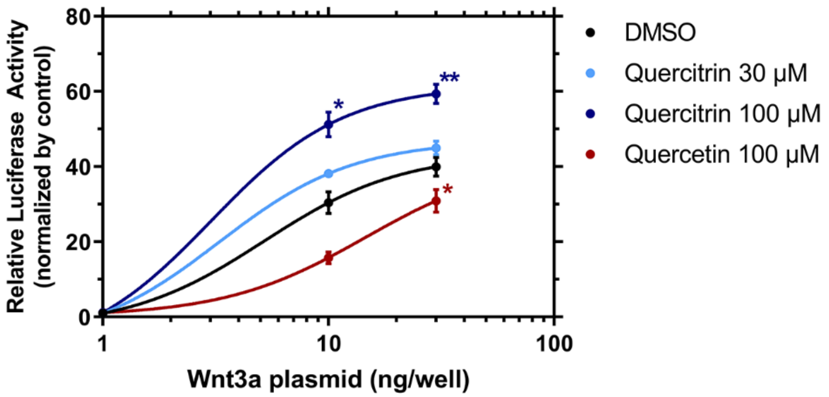

Figure S1. Quercitrin potentiates the signaling after Wnt ligand stimulation. TOPFLASH assay of HEK293T transfected with increasing amounts (1, 10, 30 ng/well) of hWnt3a plasmid. HEK293T cells were treated overnight with quercitrin or quercetin (a Wnt signaling inhibitor). (*n*=*3*, performed in triplicate, two-way ANOVA analysis considering the DMSO as the control condition followed by a Dunnett multiple comparison test. *$p<0.05$, **$p<0.01$). Error bars represent mean ± SD.

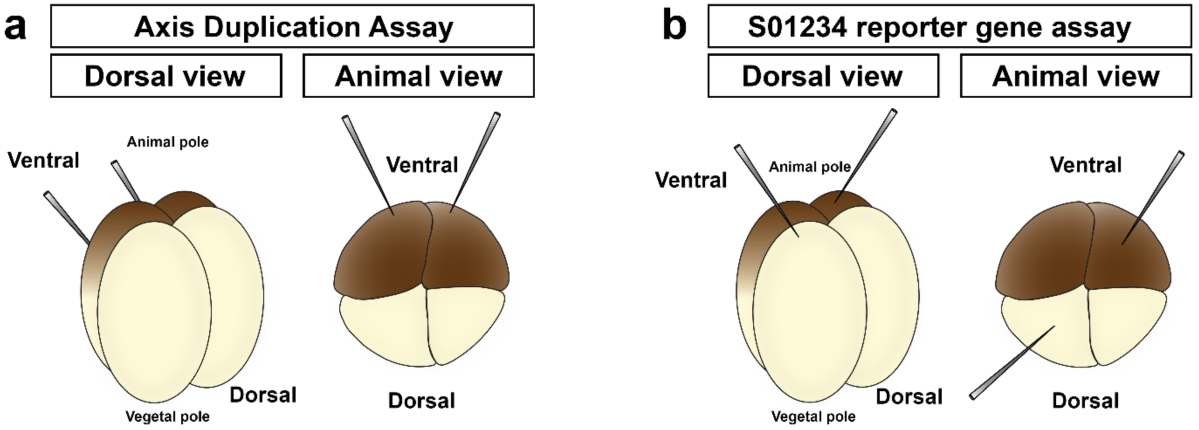

Figure S2. Injection scheme of 4-cell *Xenopus laevis* embryos. (**a**) For the Axis Duplication Assay, the ventral blastomeres were equatorially injected. (**b**) For the S01234 reporter gene assay, we injected one ventral and one dorsal blastomeres.

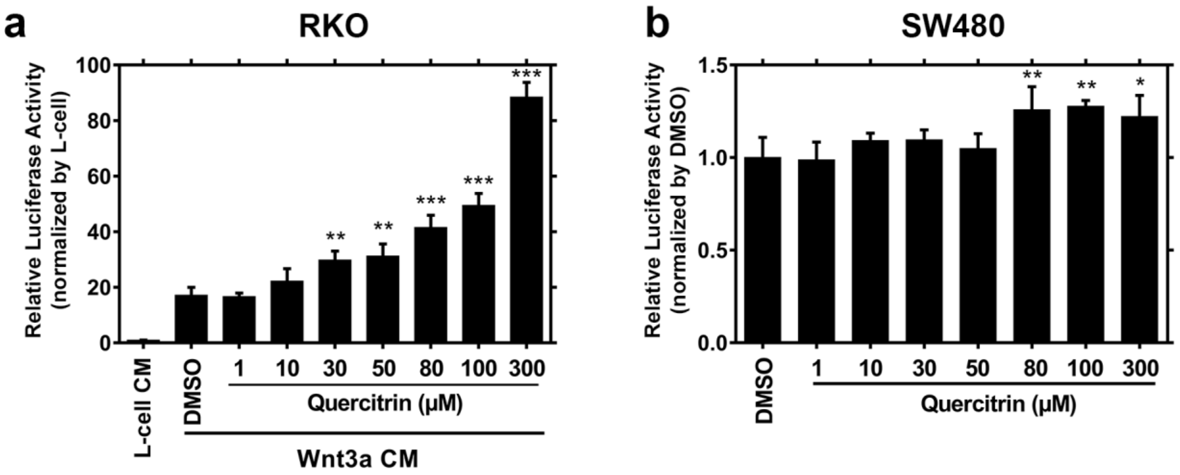

Figure S3. Quercitrin does not potentiate the Wnt signaling in the SW480 tumoral cell line. (**a**) RKO B/R cell line was treated with L-cell CM, Wnt3a CM, the vehicle DMSO, or increasing concentrations of quercitrin (n=3, performed in triplicate, one-way ANOVA followed by Dunnett's multiple comparisons test, **$p < 0.01$, ***$p < 0.001$). Error bars denote mean ± SD. (**b**) SW480 B/R reporter gene cell lines were treated with DMSO or increasing concentrations of quercitrin (n=3, performed in triplicate, one-way ANOVA followed by Dunnett's multiple comparisons test, *$p < 0.05$, **$p < 0.01$). Error bars denote mean ± SD.

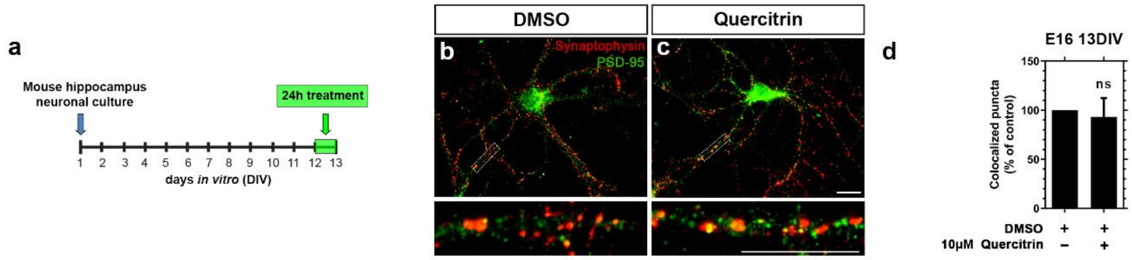

Figure S4. Quercitrin per se does not induce synaptogenesis in *vitro*. (**a**) Protocol illustration of in vitro hippocampus neuronal culture and treatment. (**b-c**) Synaptophysin and PSD-95 immunostaining of hippocampal neuronal culture. (**d**) Colocalized puncta quantification shows that 10 μM quercitrin per se does not induce synaptogenesis (*n=3*, performed in duplicate, Welch t-test statistical analysis). Error bars denote mean ± SEM. Scale bars denote 10 μm.

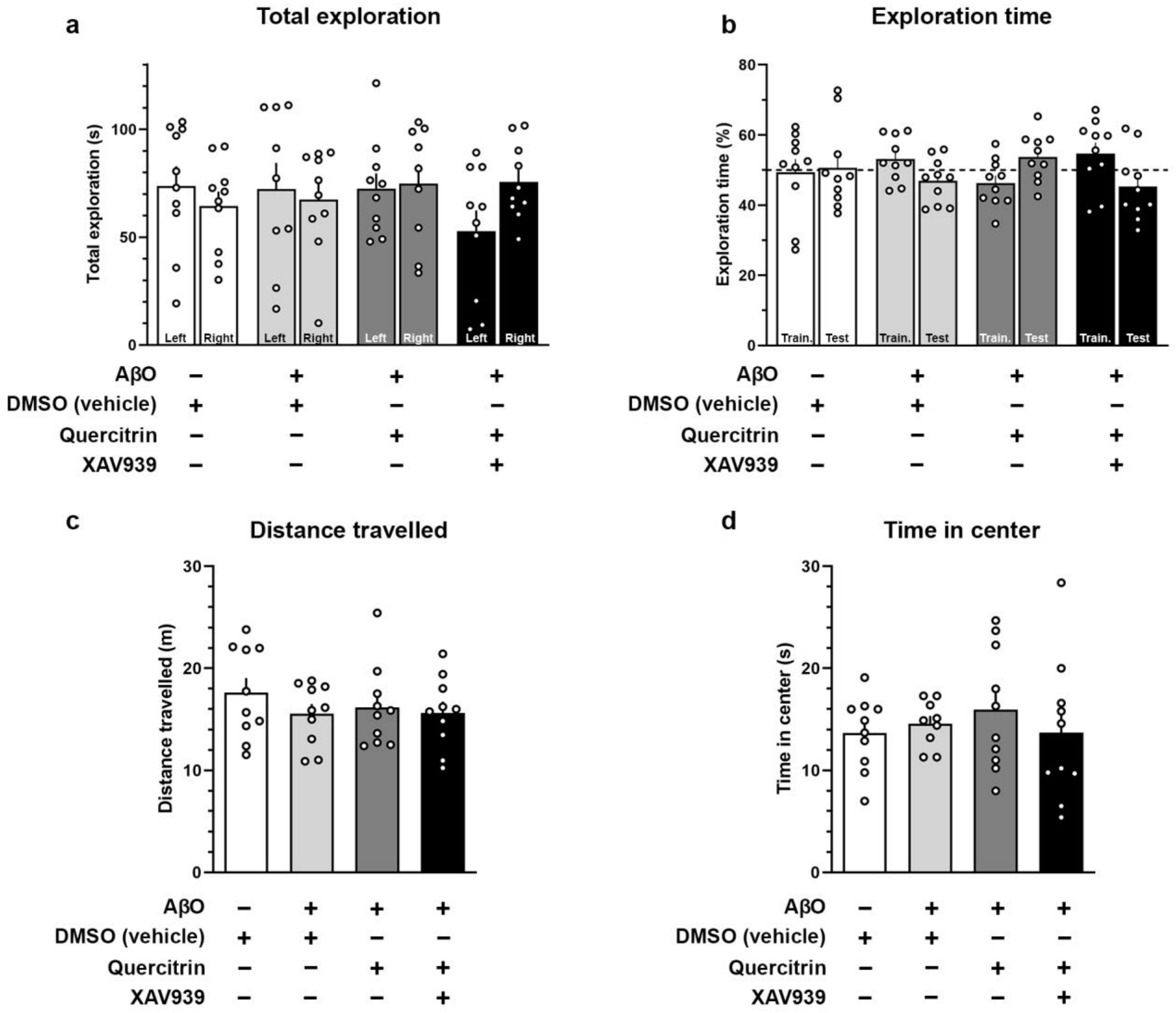

Figure S5. ICV injection does not alter the experimental exploration time of injected mice. (**a**) Total exploration time (s) of the left and right objects in the novel object recognition (NOR) training. (**b**) Exploration time (% of total time) in both objects under training and testing conditions. (**c**) Distance travelled (m) in the Open Field analysis. (**d**) Time (s) in center during the Open Field analysis.
